# Regulation of autophagy‐mediated pathways by diet, physical activity, and sleep in Alzheimer's disease

**DOI:** 10.1002/alz.71191

**Published:** 2026-02-22

**Authors:** Ajish Ariyath, Zoe Mputhia, Christopher Dougherty, Bushra Kaleelur Rahuman, W. M. A. D. Binosha Fernando, Belinda Brown, Samantha L. Gardener, Stephanie R. Rainey‐Smith, Ralph Martins, Prashant Bharadwaj

**Affiliations:** ^1^ Centre of Excellence for Alzheimer's disease Research and Care School of Medical and Health Sciences Sarich and Patricia Neuroscience Research Institute Edith Cowan University Nedlands Western Australia Australia; ^2^ Centre for Healthy Ageing Health Futures Institute Murdoch University Murdoch Western Australia Australia; ^3^ Alzheimer Research Australia Nedlands Western Australia Australia; ^4^ School of Biomedical Science Macquarie University Sydney New South Wales Australia; ^5^ Curtin Medical School Curtin Health and Innovation Research Institute (CHIRI) Faculty of Health Sciences Curtin University Bentley Western Australia Australia

**Keywords:** Alzheimer's disease, amyloid precursor protein, amyloid beta protein, autophagy, calorie restriction, diet, exercise, fasting, lifestyle interventions, physical activity, sleep, sleep deprivation, sleep fragmentation

## Abstract

Alzheimer's disease (AD) is a progressive, age‐related, neurodegenerative disorder marked by cognitive decline, memory loss, and accumulation of amyloid beta (Aβ) plaques and tau tangles. A key feature of AD is impaired protein homeostasis, often driven by autophagy dysfunction. Autophagy, a cellular degradation and recycling process, plays a vital role in maintaining neuronal health and is increasingly recognized as a therapeutic target in AD. Lifestyle factors such as diet, physical activity, and sleep can positively influence autophagy and support cognitive function. Intermittent fasting (IF) and calorie restriction (CR) activate autophagy and promote longevity; physical activity enhances cerebral blood flow and neurotrophic signaling; and adequate sleep supports autophagic processes, while sleep deprivation disrupts them. However, excessive autophagy may be detrimental. Understanding how lifestyle modulates autophagy is essential for developing non‐pharmacological strategies to delay or prevent AD. This review explores the mechanistic links between autophagy and lifestyle interventions to support brain health in aging.

## Highlights

- Lifestyle factors modulate autophagy in AD: Diet, physical activity, and sleep significantly influence autophagy, a key cellular process disrupted in AD. However, the exact mechanisms remain unclear, and effects can vary depending on intervention type, duration, and model organism.
- Autophagy is a dynamic and context‐dependent process: Autophagy fluctuates naturally with time of day, nutritional status, and physical activity. Both overactivation and impairment can be detrimental, underscoring the need for a balanced autophagic response to maintain cellular health.
- Current models and markers have limitations: Mouse models may not fully translate to human AD biology, and commonly used autophagy markers like LC3 and p62 can be misleading without complementary techniques. Emerging human‐relevant models such as brain organoids and induced pluripotent stem cell‐derived neurons may improve translational accuracy.
- Measuring brain autophagy remains a major challenge: There are no reliable biomarkers for monitoring autophagy in the human brain, complicating efforts to evaluate lifestyle interventions. Future research must focus on identifying dynamic, brain‐specific biomarkers, and safe thresholds for autophagy activation.
- Lifestyle interventions show promise but require rigorous study design: Despite being safe and accessible, lifestyle interventions face challenges in trial design, adherence, and interpretation due to their complex, multidomain nature. Well‐powered, long‐term studies are needed to evaluate their true impact on autophagy and AD prevention.

## 1 INTRODUCTION

Dementia is an umbrella term used for a wide variety of neurodegenerative diseases associated with the progressive degeneration of the central nervous system (CNS) with loss of memory and ability to perform day to day activities.^1^ Alzheimer's disease (AD) is the most common form of dementia, and age is the strongest risk factor for dementia, with an increasing prevalence in those aged over 60 years.^2^, ^3^, ^4^ Neurodegenerative diseases are characterized by the failure of protein homeostasis, which in turn leads to protein aggregation, followed by cellular dysfunction and death. Proteostasis broadly involves the regulation of protein folding, post‐translational modifications, transport, and degradation within cells.^5^ In normal aging, there is a decline in the overall efficiency and functional capacity of the two main proteostasis pathways: ubiquitin–proteasome system (UPS) and autophagic‐lysosomal pathway (ALP; referred to as autophagy).^5^, ^6^, ^7^ Autophagy is the main housekeeping mechanism for maintaining proteostasis, and its dysfunction is implicated in multiple neurodegenerative diseases.^2^, ^5^, ^8^ Autophagy is a dynamic, evolutionarily conserved pathway that degrades and recycles long‐lived proteins and damaged organelles and clears misfolded proteins and aggregates such as amyloid beta (Aβ) and neurofibrillary tangles (NFTs) of hyperphosphorylated tau.^9^, ^10^, ^11^, ^12^, ^13^ Autophagy plays a crucial role in regulating cellular processes such as growth, differentiation, and programmed cell death. It also responds dynamically to various physiological conditions, including nutrient levels, physical activity, low oxygen (hypoxia), sleep deprivation, and oxidative stress.^2^, ^5^, ^8^

### 1.1 Pathophysiology of AD

According to the World Health Organization (WHO), more than 55 million people worldwide are living with dementia as of 2024, with AD accounting for 60% to 70% of cases. Recent projections estimate that this number will rise sharply, reaching approximately 152.8 million cases by 2050.^14^ AD is characterized macroscopically by cortical and limbic atrophy, especially in the frontal and temporal lobes, leading to ventricular enlargement and reduced brain weight.^15^, ^16^ At the cellular level, AD is characterized by Aβ plaques and tau tangles. Aβ is generated from amyloidogenic cleavage of APP by β‐ and γ‐secretases, producing aggregation‐prone Aβ42 peptides that disrupt cellular homeostasis, trigger oxidative stress, and induce neuronal death. Mutations in γ‐secretase components (presenilin 1 and presenilin 2 [PSEN1/2]) are linked to early‐onset AD.^17^, ^18^, ^19^, ^20^, ^21^, ^22^ Tau, a microtubule‐stabilizing protein, becomes hyperphosphorylated, forming NFTs that impair axonal transport and exacerbate neuronal damage. Aβ and tau pathologies are closely interlinked, contributing synergistically to AD progression.^23^, ^24^, ^25^ Understanding the intricate relationship between autophagy dysfunction and protein aggregation not only deepens our insight into AD pathogenesis but also opens new avenues for therapeutic strategies aimed at restoring autophagic balance and preventing neuronal loss.

### 1.2 Autophagy

Dysregulated autophagy is a key mechanism in neurodegenerative diseases such as AD, amyotrophic lateral sclerosis, Parkinson's disease, and Huntington's disease, where toxic protein starts aggregating over time.^26^, ^27^, ^28^ Autophagy is classified into three main types, depending on the specific pathway used to deliver intracellular components to the lysosome for degradation.^10^, ^13^ Macroautophagy, microautophagy, and chaperone‐mediated autophagy (CMA) are the three main autophagy pathways.

#### 1.2.1 Macroautophagy

**FIGURE 1:**
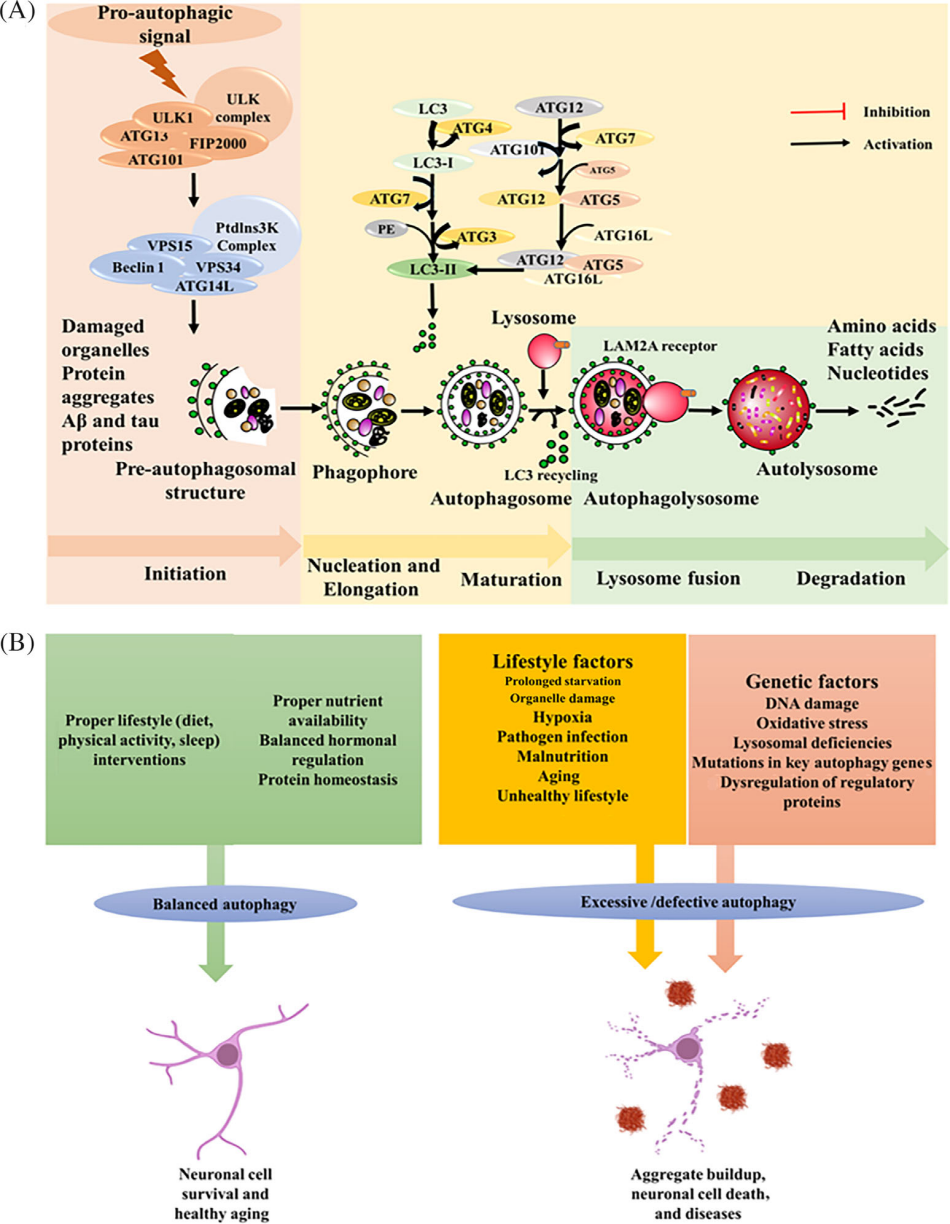
Autophagic pathway and its balance. (A) Pro‐autophagic signaling activates the ATG machinery, including key regulators like ULK1 and the Beclin‐1 complex. This activation initiates the four sequential stages of autophagy initiation, nucleation and elongation, maturation, lysosome fusion, and degradation. (B) Autophagic pathway and its balance. Proper lifestyle, nutrient availability, balanced hormonal regulation, and protein homeostasis lead to a balanced autophagy, crucial for neuronal cell survival and healthy aging. An unbalanced lifestyle coupled with other genetic factor leads to excessive/defective autophagy, resulting in aggregate buildup, increased neuronal cell death, and disease. ATG, autophagy regulated genes; ULK1, Unc‐51 like autophagy activating kinase 1.

Macroautophagy is primarily a non‐selective bulk degradation process activated by starvation or nutrient deprivation. In this pathway, cargo is sequestered into autophagosomes, which then fuse with lysosomes for degradation.^29^ Macroautophagy (referred to hereafter as autophagy) involves the formation of double membrane autophagosomes that engulf cytoplasmic material and deliver it to lysosomes for degradation.^10^, ^12^ It begins with the activation of the ULK1 complex (ULK1, Atg13, Atg101, FIP200), which initiates phagophore formation.^13^, ^30^ Expansion of the phagophore is driven by the class III PI3K complex (Vps34, Vps15, Beclin‐1, Atg14L), enabling membrane nucleation.^13^, ^30^, ^31^ During elongation, LC3 undergoes Atg7/Atg3‐mediated lipidation to LC3‐II, allowing cargo recruitment and autophagosome maturation (Figure 1A).^31^ Mature autophagosomes fuse with lysosomes through SNARE proteins (Syntaxin‐17, SNAP29, VAMP8) and Rab7, where degradation occurs under acidic conditions maintained by v‐ATPase.^30^, ^31^, ^32^, ^33^ Each stage is tightly regulated, and any defects in the process contribute to the accumulation of misfolded proteins such as Aβ and tau, leading to neurodegeneration.

#### 1.2.2 Microautophagy

Microautophagy is a lysosomal degradation process in which cytoplasmic material is directly internalized through lysosomal membrane invagination.^31^, ^34^ Unlike macroautophagy, it does not require autophagosome formation and can function in both selective and non‐selective modes depending on cellular conditions.^34^ Several other forms of selective autophagy exist, each involving a core set of machinery and specific components. These are distinguished by unique names based on their targeted substrates, such as nucleophagy (nucleus), mitophagy (mitochondria), pexophagy (peroxisomes), lysophagy (lysosomes), xenophagy (bacterial and viral pathogens), aggrephagy (protein aggregates), and endoplasmic reticulum (ER)‐phagy, among many others.^35^ Overall, mitophagy is the most thoroughly investigated, closely followed by other recognized forms such as ER‐phagy and aggrephagy. The elimination of damaged mitochondria (mitophagy) is essential for cellular health and has been associated with numerous diseases.^36^ However, the involvement of microautophagy in human health and disease remains largely unknown due to the restricted number of tools available for its investigation.^37^ A major limitation in microautophagy research is the absence of specific molecular markers, live‐cell imaging tools, and suitable genetic models, which restrict our understanding of its role in human health and disease. Further research is needed to elucidate the molecular mechanisms of microautophagy, its tissue‐specific roles, and potential involvement in neurodegenerative diseases, including AD.

#### 1.2.3 Chaperone‐mediated autophagy

Another major form of autophagy is CMA, a selective lysosomal pathway in which soluble proteins containing a KFERQ motif are recognized by cytosolic chaperones (Hsc70/Hsp70) and delivered to the lysosomal receptor LAMP2A for degradation.^9^, ^10^, ^38^, ^39^, ^40^ Although CMA contributes to protein quality control, its specific role in AD remains unclear due to limited tools for measuring CMA activity in mammalian neurons.

### 1.3 Regulation of autophagy

Autophagy is vital for maintaining cellular homeostasis.^39^ Pro‐autophagic signaling activates the autophagy regulated genes (ATG) machinery, including key regulators like ULK1 and the Beclin‐1 complex.^10^, ^12^ This activation initiates the four sequential stages of autophagy initiation, nucleation and elongation, maturation, lysosome fusion, and degradation (Figure 1A). Autophagic regulation is mainly carried out by two key nutrient sensors, such as mechanistic target of rapamycin (mTOR) and adenosine monophosphate (AMP)‐activated protein kinase (AMPK).^38^

mTOR is a serine/threonine kinase belonging to the phosphatidylinositol 3‐kinase‐related kinase (PIKK) family and plays a central role in cellular nutrient sensing and metabolic regulation.^41^, ^42^ mTOR acts as a key negative regulator of autophagy by interacting with multiple signaling proteins to inhibit catabolic processes and stimulate protein synthesis.^5^, ^38^, ^40^ mTOR exists in two distinct complexes with different functions and associated proteins. mTOR complex 1 (mTORC1) includes mTOR, Raptor, mLST8, and PRAS40, while mTORC2 is composed of mTOR, Rictor, mLST8, and Sin1.^38^, ^43^ mTORC2 primarily regulates kinases such as Akt/protein kinase B (PKB) and protein kinase C (PKC) during autophagy.^38^ mTORC1 inhibits autophagy initiation by phosphorylating ULK1 and Atg13, two key proteins required for the early stages of autophagosome formation.^35^, ^38^ During normal physiological conditions, mTORC1 suppresses autophagy by inhibiting ULK1, a key initiator of the autophagic process, and by phosphorylating transcription factor EB (TFEB), a master regulator of genes involved in autophagy and lysosomal biogenesis.^42^, ^44^, ^45^

mTORC1 also has an important role in protein synthesis by the downstream activation of p70S6 kinase or S6K.^38^ mTORC1 promotes protein synthesis by activating S6K, which in turn negatively regulates autophagy. Interestingly, S6K activity may also contribute to autophagosome maturation under certain conditions. S6K is one of the negative regulators of autophagy as its main function is ribosomal protein synthesis. Some reports suggest an upregulation of S6K during autophagy, which can be explained by the fact that S6K is primarily involved in protein synthesis, which may be used in the process of autophagy initiation and maturation process.^38^, ^44^ As reports suggest, excessive autophagy or self‐eating can lead to cell death. Therefore, a negative regulator like S6K may help maintain autophagic balance by moderating the inhibitory effect of mTORC1. This moderation prevents overactivation of autophagy, protecting cells from autophagy‐induced damage while still allowing necessary cellular clearance and homeostasis.^38^, ^46^ While autophagy typically protects against cellular stress, excessive activation/defect can lead to neuronal cell death and aggregate buildup, highlighting the need for precise regulation (Figure 1B).

The AMPK pathway serves as an energy sensor and acts as a positive regulator of autophagy, especially when mTORC1 is inhibited under energy‐deprived conditions.^41^ AMPK is a serine/threonine protein kinase that has been conserved throughout evolution, which functions as a cell's energy sensor.^47^, ^48^ It is an essential component in upregulating catabolic processes, such as glucose metabolism and uptake, and inactivating anabolic processes, such as carbohydrate, lipid, and protein biosynthesis.^49^ These effects in turn contribute to the maintenance of homeostasis of the internal environment and energy balance.^50^ Under cellular energy scarcity, the nutrient sensor AMPK is activated by the decreased levels of ATP/AMP ratio. Additionally, active AMPK inhibits the mTORC activity through Rheb proteins, activating the TSC1/2 complex.^38^, ^41^ AMPK also inhibits HMGCoA reductases and prevents cholesterol and fatty acid synthesis by inhibiting Acetyl‐CoA carboxylase (ACC) by phosphorylation.^51^, ^52^ AMPK has also been shown to activate ULK1 in fatty acid catabolism by activating adipose triglyceride lipase.^52^, ^53^

Under nutrient‐rich conditions, ULK1 is bound to mTOR, which prevents the initiation of the autophagy process.^54^, ^55^ Conversely, during nutrient deprivation, mTOR detaches itself from the ULK1 complex, which activates the autophagy process.^55^ During fasting, AMPK plays a key role in activating autophagy by directly phosphorylating ULK1, a central kinase in the autophagy initiation complex, and concurrently inhibiting mTORC1, a major autophagy suppressor. A pivotal study by Kim et al. revealed that AMPK and mTORC1 exerted opposing regulatory effects on ULK1, highlighting the integration of metabolic signals at the level of autophagy initiation. Specifically, AMPK activates ULK1 by phosphorylating it at Ser317 and Ser777, thereby promoting autophagy under energy‐stressed conditions. In contrast, under nutrient‐rich conditions, elevated mTORC1 activity inhibits autophagy by phosphorylating ULK1 at Ser757, a modification that disrupts the interaction between AMPK and ULK1, effectively suppressing ULK1 activation. Furthermore, the study demonstrated that inhibition of mTORC1 using rapamycin enhances AMPK‐mediated phosphorylation of ULK1, reinforcing the interplay between these nutrient‐sensing pathways. This intricate regulatory network between AMPK, mTORC1, and ULK1 represents a major advancement in our understanding of how cells fine‐tune autophagy in response to metabolic status and stress signals.^51^, ^52^, ^54^

While the core autophagy machinery is primarily regulated by non‐transcriptional nutrient sensors such as mTOR and AMPK, autophagy can also be enhanced through transcriptional regulation, notably via the Forkhead box O3 (FoxO3) transcription factor^56^, ^57^ (Figure 1A). Studies on protein breakdown during muscle atrophy led researchers to uncover a transcription‐dependent mechanism for mammalian cells via FoxO3.^56^, ^57^ FoxO3 induces numerous autophagy genes, including ATG4B, ATG12, BNIP3, BNIP3L, Beclin‐1, GABARAPL1, LC3B, ULK2, and VPS34.^58^ Of these genes, gene transcription is induced when FoxO3 binds specifically to the promoters of ATG12, BNIP3, BNIP3L, GABARAPL1, and LC3B.^58^ TFEB, a basic helix–loop–helix‐leucine‐zipper (bHLH‐Zip) protein, regulates the expression of target genes bearing the Coordinated Lysosomal Expression and Regulation (CLEAR) motif, thereby modulating autophagy and lysosomal biogenesis. In addition to regulating autophagy and lysosomal biogenesis, TFEB plays a role in selective autophagy and lysosomal exocytosis, making it a potential therapeutic target in diseases involving lysosomal dysfunction.^59^ Together, these insights underscore the complexity and precision of autophagy regulation, which is governed by both nutrient‐sensing kinases like mTOR and AMPK and transcriptional regulators such as FoxO3 and TFEB. The dynamic interplay between these pathways ensures cellular adaptation to metabolic stress and nutrient availability.

### 1.4 Autophagy dysfunction in AD

Autophagy, a key cellular proteostasis mechanism, is closely linked with the pathogenesis of protein misfolding disorders such as AD.^60^, ^61^ Early studies identified dysfunction in the endosomal lysosomal system as a critical regulator of amyloid precursor protein (APP) processing, a contributor to AD pathogenesis and Aβ generation. Notably, the endocytic pathway was found to be hyperactive in vulnerable neuronal populations, with early endosomes displaying significant enlargement in the AD brain. Aβ was observed within these enlarged, Rab5‐positive endosomes, implicating early endosomal dysfunction in amyloidogenesis.^62^, ^63^ Nixon et al. published one of the first studies demonstrating a clear link between autophagic dysfunction and neurodegeneration, highlighting the critical role of autophagy in maintaining neuronal health.^64^ Using immunogold staining and electron microscopy, they showed a significant accumulation of autophagosomes in the frontoparietal cortex of AD patients compared to cognitively unimpaired older adults. These findings suggest that enhancing autophagy could be a promising therapeutic strategy for treating AD and other neurodegenerative disorders.^64^ In line with these findings, Pickford et al. reported significantly reduced levels of Beclin‐1, an essential autophagy‐regulating protein in AD brain tissues at both the transcript and protein levels.^65^ Other studies expanded this perspective, demonstrating that autophagic dysfunction contributes not only to Aβ pathology but also to tau accumulation and neurofibrillary tangle formation.^66^, ^67^, ^68^ In AD, the impaired clearance of Aβ and hyperphosphorylated tau is closely associated with defects in the autophagy‐lysosomal pathway. Genetic mutations and polymorphisms that affect autophagy‐related components exacerbate this dysfunction. Notably, mutations in PSEN1, a component of the γ‐secretase complex, not only alter APP processing but also disrupt lysosomal acidification and autophagosome clearance.^69^, ^70^

Autophagy is tightly interconnected with other pathological mechanisms in AD, including chronic neuroinflammation and oxidative stress. In microglia, autophagy constrains inflammasome activity; targeting NLRP3 shows that enhancing microglial autophagic degradation limits IL‐1β–driven neuroinflammation in AD models.^71^, ^72^ Impaired autophagy is associated with increased reactive oxygen species (ROS) and organellar damage; cells and patient‐derived lines from AD display elevated mitochondrial ROS alongside defects in autophagy/lysosomal degradation.^73^, ^74^ Conversely, oxidative stress can directly block autophagosome‐lysosome fusion, for example, by disrupting SNARE machinery (VAMP8) or lowering STX17, leading to accumulation of dysfunctional autophagic vesicles.^75^, ^76^ Thus, autophagy is both regulated by and protective against inflammatory and oxidative mechanisms, underscoring the importance of restoring autophagic balance in AD.

Mutations in PSEN1 have been implicated not only in aberrant Aβ production but also in autophagic dysfunction. PSEN1 is essential for the proper acidification and maturation of lysosomes. Studies have demonstrated that PSEN1 mutations impair lysosomal proteolysis by disrupting the proper targeting of vacuolar‐type H^+^‐ATPase (v‐ATPase) to lysosomes. This defect leads to impaired autophagosome clearance, independent of PSEN1’s γ‐secretase function, highlighting its multifaceted role in maintaining cellular homeostasis.^69^, ^70^ Supporting these findings another study demonstrates that PSEN1 deficiency impairs autophagy in human neural stem cells by reducing extracellular signal‐regulated kinase/cAMP response element‐binding protein (ERK/CREB) signaling, a γ‐secretase‐independent pathway.^77^ Together, these findings highlight the pivotal role of PSEN1 in regulating autophagy through both lysosomal function and intracellular signaling pathways. Its dysfunction is strongly associated with impaired cellular homeostasis and is a key contributor to the pathogenesis of neurodegenerative diseases such as AD.

Cathepsin D and PRKAG2 are emerging as important regulators of the autophagy‐lysosomal pathway, with growing evidence linking their dysfunction to neurodegenerative diseases like AD. Cathepsin D, a major lysosomal aspartic protease, plays a critical role in autophagic degradation by breaking down aggregated and misfolded proteins. Genetic polymorphisms or reduced activity of cathepsin D have been associated with impaired lysosomal function and a higher risk of AD.^78^ In addition, mutations in PRKAG2, a gene encoding the regulatory subunit of AMPK, impair cellular energy sensing and autophagy activation. Studies have also revealed genes that are involved in the autophagy dysfunction observed in AD. Furthermore, findings from our lab demonstrated that the expression of PRKAG2 gene was elevated threefold in the frontal cortex and hippocampus of AD brains compared to controls. Moreover, PRKAG2 protein levels were associated with increased Aβ accumulation in the brain.^79^ A study in yeast additionally demonstrated that autophagy was activated by Aβ42 expression and co‐expression of the PRKAG2 homolog, SNF4, significantly decreased Aβ42 aggregate levels, and autophagic activity in controls.^80^ These two studies therefore suggest that elevated levels of Aβ accumulation in the AD brain may result in increased PRKAG2 expression and autophagy activation. Another protein, phosphatidylinositol binding clathrin assembly protein (PICALM) is a crucial protein involved in clathrin‐mediated endocytosis and intracellular trafficking, processes that intersect with autophagy and lysosomal function. Abnormal cleavage of PICALM, whose loss of function has a detrimental effect on autophagy, is increased in AD.^81^ The loss of function of PICALM on autophagy results in erroneous autophagosome formation and maturation.^82^ Overall, these findings show that increased autophagy activation and expression of autophagy pathway genes could be a mitigating response to increased Aβ accumulation in the AD brain. Together, these genetic factors underline the critical role of autophagy in the development and progression of AD and other proteinopathies, highlighting the need for therapeutic strategies aimed at restoring autophagic function.

### 1.5 Mitochondrial dysfunction and autophagy in AD

The interaction between mitochondrial dynamics and autophagy, previously viewed as a crucial homeostatic mechanism for preserving cellular integrity, is now increasingly acknowledged as a primary contributor to neurodegenerative pathology in AD.^83^ A key pathological feature of AD is mitochondrial dysfunction, which is closely related to impaired autophagy.^84^, ^85^, ^86^, ^87^ Redox homeostasis, calcium regulation, and neuronal energy metabolism all depend on mitochondria. In AD, synaptic failure and neuronal loss are mediated by mitochondria that have altered morphology, decreased oxidative phosphorylation, and excessive ROS production.^88^ The accumulation of dysfunctional mitochondria is mostly caused by impaired mitophagy, a process that eliminates damaged mitochondria selectively and results in oxidative stress and metabolic dysregulation.^83^ Studies have shown that changes in the PINK1/Parkin‐mediated mitophagy pathway are important for the development of AD, as ineffective mitochondrial clearance results from decreased Parkin translocation and PINK1 stabilization on damaged mitochondria.^89^, ^90^ As a result, abnormal mitochondria build up and release too many ROS and apoptotic factors, which worsen tau hyperphosphorylation and Aβ aggregation.^89^, ^90^, ^91^ Neuronal degeneration is also made worse by impaired mitochondrial dynamics, which are characterized by an imbalance between fusion and fission. There have been reports of protein dysregulation in AD, including dynamin‐related protein 1 (Drp1) and mitofusins (Mfn1/2), which results in mitochondrial fragmentation and synaptic dysfunction.^92^, ^93^ Emerging studies suggest that restoration of mitochondrial dynamics and enhancement of mitophagy could serve as a potential therapeutic approach to counteract neurodegeneration in AD. To preserve neuronal homeostasis, autophagy and mitophagy are crucial for mitochondrial quality control, removing faulty or unnecessary organelles.

### 1.6 Pharmacological treatment strategies for AD

AD treatment currently involves several US Food and Drug Administration (FDA)‐approved drugs aimed at managing both cognitive symptoms (such as memory loss, impaired thinking, and language deficits) and non‐cognitive symptoms (including behavioral and psychological changes). FDA‐approved drugs includes cholinesterase inhibitors, glutamate regulators, orexin receptor antagonists for treating insomnia, and antipsychotics for managing behavioral disturbances.^66^, ^94^, ^95^, ^96^, ^97^ Disease‐modifying therapies focus on Aβ and tau pathologies.^98^, ^99^ Aβ is targeted via immunization strategies and secretase inhibitors.^100^, ^101^, ^102^ Recently approved monoclonal antibodies, such as donanemab and lecanemab, reduce Aβ plaques but are associated with adverse effects like amyloid‐related imaging abnormalities.^103^, ^104^ Tau‐targeting approaches, including kinase inhibitors and immunotherapy, have not yielded significant clinical benefits.^25^, ^105^, ^106^, ^107^, ^108^ Given that impaired autophagy contributes to the aggregation of unwanted proteins like Aβ and tau in AD, its therapeutic activation through mTOR‐dependent (e.g., rapamycin) and mTOR‐independent mechanisms (e.g., nilotinib, GTM‐1, latrepirdine, trehalose) is a promising strategy.^26^, ^51^, ^109^, ^110^, ^111^, ^112^, ^113^, ^114^, ^115^ However, concerns such as immunosuppression, age‐related variability, and potential lysosomal dysfunction remain challenges.^79^, ^105^, ^106^, ^107^, ^108^ Emerging evidence also suggests that lifestyle modifications, such as regular dietary interventions, physical activity, cognitive engagement, and sleep, may play a crucial role in preventing or slowing AD progression.^116^, ^117^, ^118^, ^119^

### 1.7 Non‐pharmaceutical treatments for AD

AD poses a growing public health challenge, with rising prevalence due to global aging. Therefore, developing approaches that are effective in lowering the financial and social expenditures, preserving the economic productivity of family carers, and minimizing the impact on the healthcare system will be very beneficial. It has been revealed that most age‐related dementias, especially AD, co‐exist with multiple comorbid conditions, mainly cerebrovascular diseases (CVDs).^120^, ^121^ The broader implication is that systemic vascular factors are risk factors for developing AD and related dementias. Major modifiable lifestyle factors for the onset of AD (e.g., diet, level of education, alcohol consumption, leisure, physical, sleep, and social activities), as well as traditional CVD risk factors (e.g., dyslipidemia, type II diabetes, carotid atherosclerosis, hypertension, smoking, and hypercholesterolemia), have therefore been the focus of epidemiological, preclinical, and interventional studies.^118^, ^121^, ^122^, ^123^, ^124^, ^125^, ^126^ While pharmaceutical treatments offer short‐term relief, lifestyle interventions have shown greater potential for long‐term prevention. Autophagy, a key cellular housekeeping mechanism, is emerging as a critical target in AD prevention. A recent review by Ortega et al. highlights the close relationship between lifestyle factors and autophagy, emphasizing diet, physical activity, sleep patterns, and environmental conditions influence on the regulation of the autophagic process.^119^ Non‐pharmacological strategies focused on lifestyle, especially diet, physical activity, and sleep, offer promising avenues to delay or prevent AD.^127^, ^128^ Yet, few studies explore the molecular mechanisms behind these benefits. Understanding how lifestyle interventions regulate autophagy may enhance prevention strategies and improve clinical trial outcomes through better biomarker use and disease modeling. Autophagy is a complex mechanism, and understanding its pathway for adopting proper preventive strategies is crucial for a successful outcome. A balanced autophagic pathway is essential for cellular homeostasis, preventing the accumulation of toxic aggregates while avoiding excessive self‐digestion (Figure 1B).

Overall, non‐pharmacological intervention and lifestyle changes focusing on diet, physical activity, and sleep could unlock new avenues for prevention and delaying of AD.^129^, ^130^, ^131^ The AD processes start many years before clinical symptoms manifest, so recent attention has been given to delaying or preventing the onset of AD at an earlier stage of life by the introduction of lifestyle interventions. However, only a handful of studies attempted to dissect the molecular pathways underlying the benefits of such interventions.^130^, ^131^ It is important to understand the mechanisms in which lifestyle factors regulate homeostasis mechanisms such as autophagy to develop more effective treatment and risk reduction strategies for AD and related neurodegenerative diseases. Research into basic disease mechanisms can have immense benefit for development of strategies to reduce risk or improve prevention. Moreover, clinical trials will have improved rates of success when disease mechanisms and their relevant clinical biomarkers are well defined.

### 1.8 Diet and autophagy

Various dietary interventions, including calorie restriction (CR) and intermittent fasting (IF) and ketogenic, Mediterranean, plant‐based, and polyphenol‐rich diets, modulate key nutrient sensing pathways (e.g., mTOR inhibition; activation of AMPK, SIRT1, and TFEB) to enhance autophagic flux.^132^, ^133^, ^134^, ^135^ However, clinical translation is challenged by long‐term dietary adherence, individual variability in dietary response, and feasibility in older adults with cognitive impairment. Dietary modulation of autophagy presents a promising non‐pharmacological strategy to delay or prevent AD progression, but further clinical trials are needed to validate long‐term effects and adherence feasibility in older adults.

### 1.9 Fasting and CR

#### 1.9.1 Mechanism of fasting and autophagy

**FIGURE 2:**
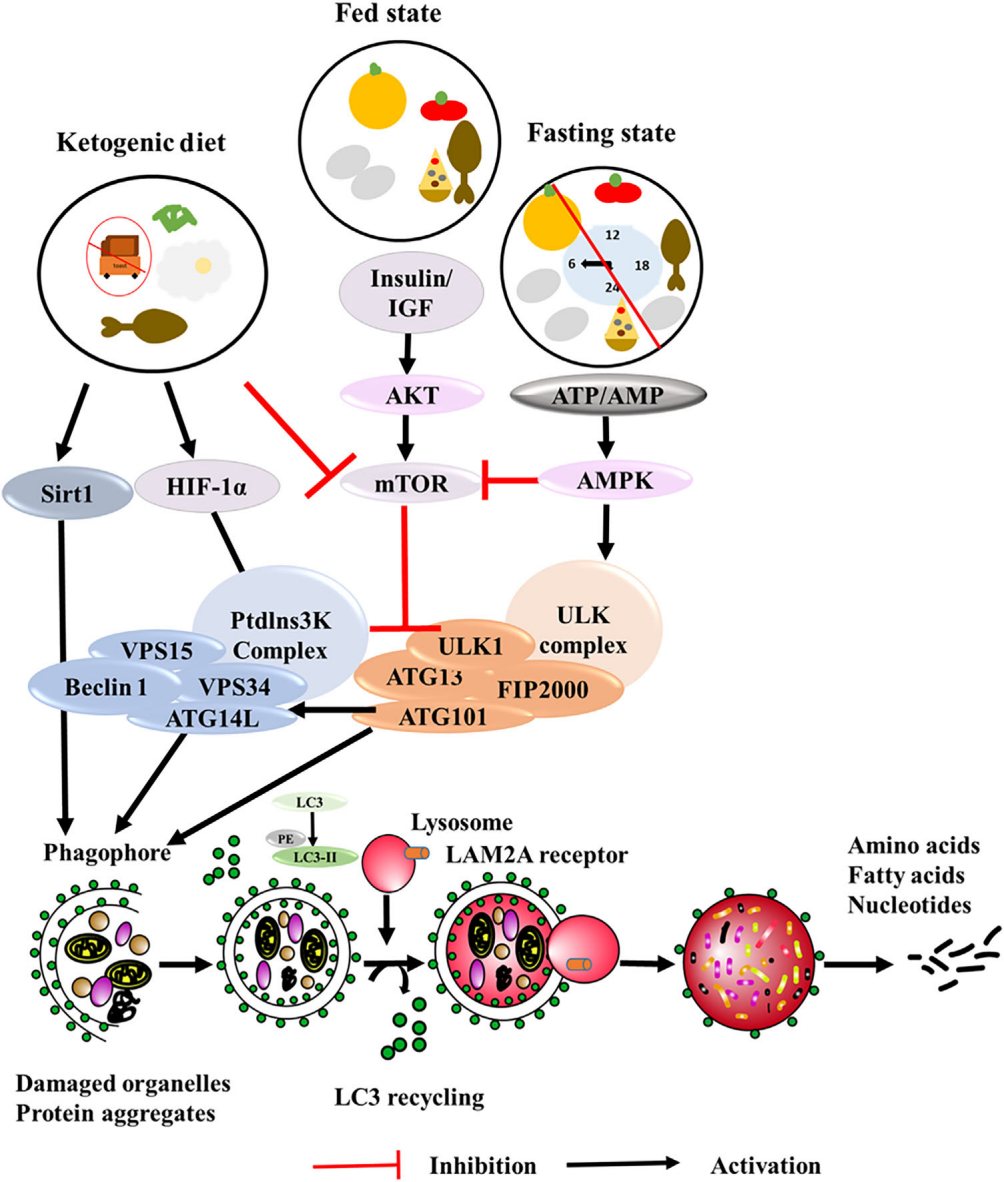
Impact of diet on modulation of autophagic pathway. Fasting through intermittent fasting (IF) or caloric restriction (CR) induces autophagy by activating AMPK and inhibiting mTOR, hence facilitating cellular recycling, while the fed state reduces autophagy via mTOR activation, based on nutrition and energy availability. A ketogenic diet activates Sirt1 and HIF‐1α activating the autophagic pathway and indirectly by inhibiting mTOR. A proper dietary regime is key for preserving cellular integrity and averting age‐related disorders, including neurodegenerative diseases. Aβ, amyloid beta; AMPK, adenosine monophosphate‐activated protein kinase; ATG, autophagy regulated genes; CR, caloric restriction; IF, intermittent fasting; IGF, insulin‐like growth factor; mTOR, mechanistic target of rapamycin; ULK1, Unc‐51 like autophagy activating kinase 1.

Fasting triggers a metabolic shift from glycolysis to gluconeogenesis, enabling cells to utilize alternative energy sources like ketone bodies, which may enhance cognitive function and brain resilience^55^, ^136^, ^137^ (Figure 2). It is considered one of the most potent activators of autophagy, promoting cellular homeostasis through the clearance of toxic metabolites and misfolded proteins.^55^, ^136^, ^137^ At the molecular level, fasting modulates key nutrient‐sensing pathways. It downregulates the insulin/IGF‐1 and mTOR pathways and upregulates AMPK, an energy sensor essential for mitochondrial function and autophagy induction.^55^, ^112^, ^136^, ^137^ These shifts support protein homeostasis and neuroprotection. IF and CR also elicit neuroendocrine responses, adjusting levels of glucose, insulin, and amino acids, which further suppress IGF‐1 and mTOR signaling to enhance autophagic activity.^108^, ^136^, ^137^

#### 1.9.2 Animal studies

Experimental studies across model organisms, from yeast to mammals consistently demonstrate that IF and CR enhance autophagy, increase stress resistance, and extend lifespan. In *Saccharomyces cerevisiae* (yeast), nutrient deprivation suppresses TORC1 and Sh9 signaling, doubling lifespan and improving resilience to oxidative stress and Aβ toxicity.^113^, ^138^, ^139^, ^140^ In Caenorhabditis *elegans* (worms), nutrient scarcity elevates autophagy markers and extends lifespan,^138^, ^141^ while in *Drosophila melanogaster* (fly), amino acid starvation activates autophagy via TOR and PI3K signaling, promoting longevity.^142^ Ulgherait et al. used a drosophila model under an intermittent time restricted fasting regime to show a strong association between the autophagy process and circadian genes. Autophagy genes ATG1 and ATG8 (the mammalian homologues of ULK1 and LC3, respectively) in drosophila were regulated with a high expression at night and low expression during the daytime.^143^ In rodent models, both IF and CR have been shown to activate autophagy, improve cognitive outcomes, and protect against neurodegeneration (Table 1). These findings, along with changes in autophagy‐related markers in response to dietary interventions, are summarised in Table 1 and further detailed below.

**TABLE 1:** Autophagy‐related expressions in the nervous tissues following different diet regimes.

Diet type	Study	Rodent model	Duration	Target nervous tissue	AD‐ and autophagy‐related expressions of interest findings
**Fasting diets**					
IF	Madorsky et al.^145^	TrJ mice	5 months	Peripheral nerves	↑ Atg7, LC3 (autophagosome formation)↓ mTOR, pS6, p62
Short‐term fasting	Alirezaei et al.^148^	GFP‐LC3 mice	24/48 h	Cortical nerves	↑ LC3 (autophagosome formation) ↓ mTOR, pS6
Short‐term fasting	Chen et al.^147^	5xFAD mice	0 to 48 h	Cortical nerves	↑ LC3 (autophagosome formation)
CR	Rangaraju et al.^146^	Fischer 344 rat	Age‐associated (8, 18, 29, 38 months)	Peripheral nerves	(In vitro) Young rats: ↑ Atg7, LC3II Old rats: ↑ Atg7; (in vivo) ↑ LAMP1, Atg7; ↓ pS6, S6
CR (low calorie)	Dong et al.^150^	C57/BL6 mice	10 months	Hippocampal neuron	↑ Beclin‐1, cathepsin B, LC3 ↓ mTOR, p62
CR	Gregosa et al.^144^	PDAPP‐J20 mice	6 weeks	Neuroglia	↑ LC3 ↓ Aβ
CR	Müller et al.^151^	APPswe/PS1delta9 (tg) mice	68 weeks	Hippocampus	↑ LC3BII, p62; ↓ Aβ
IF	Ntsapi and Loos^155^	GFP‐LC3 mice	48 h	Cortex, cerebellum, and hippocampus	↑ LC3II, p62, ATG5, LAMP2A
CR (short term)	Liu et al.^149^	C57/BL6 mice	30 days	Hippocampus	↑ LC3B, Beclin‐1; ↓ mTOR
CR	Ferreira‐Marques, M., et al.^152^	Female Wistar rats	16 to 24 weeks	Cortical neurons	↑ LC3II ↓ p62, phospho‐MTOR
CR	Bensalem et al.^153^	NL‐G‐F mice	4 months	Cerebral cortex	↓ mTORC1
**High fat/calorie/cholesterol diets**					
High‐fat diet	Wen et al.^156^	C57BL/6 mice	6 months		↑ Beclin‐1, LC3II/LC3I
High calorie	Dong et al.^150^	C57/BL6 mice	10 months	Hippocampus	↑ mTOR, S6K
High cholesterol	Wang et al.^157^	C57BL/6J mice	28 days	Cerebral cortex	↑ t‐tau, p‐tau, mTOR ↓ Beclin‐1, LC3B
**Ketogenic diets**					
Ketogenic diet	McDaniel et al.^167^	Sprague‐Dawley rats	2 weeks	Hippocampus and frontal cortex	↓ mTOR, pS6, pAkt,
Ketogenic diet	Liśkiewicz et al.^177^	9‐week‐old male mice	4 weeks	Hippocampus and liver	↑ LC3II
Ketogenic diet	Liśkiewicz et al.^178^	C57BL/6N male mice	4 weeks	Hippocampus and liver	↑ Beclin‐1, LC3II/LC3I

*Note*: ↑, increased expression in autophagic markers, ↓, decreased expression in autophagic markers.

Abbreviations: Aβ, amyloid beta; AD, Alzheimer's disease; CR, caloric restriction; IF, intermittent fasting; KD, ketonic diets, mTOR (mTORC1), mechanistic target of rapamycin (complex 1); p‐tau, phosphorylated tau.

Gregosa et al. demonstrated the potential involvement of CR‐induced glial autophagy in Aβ clearance in an AD mouse model.^144^ Studies have also demonstrated that short‐term fasting in mice (food restricted for 24 or 48 h) leads to a dramatic upregulation in neuronal autophagy,^145^, ^146^ as well as increased autophagosome formation.^147^, ^148^ Additionally, short‐term CR over 30 days has been shown to exert neuroprotective effects following mild traumatic brain injury by enhancing autophagy and inhibiting astrocyte activation, thereby reducing neural damage.^149^ Long‐term CR has been shown to modulate autophagy and improve age‐related cognitive function and hippocampal integrity,^150^ attenuate Aβ pathology in AD mouse models,^151^ and stimulate autophagy in cortical neurons via neuropeptide Y and ghrelin receptor pathways.^152^ Additionally, dietary protein levels under CR conditions influence mTOR signaling and Aβ accumulation, further implicating nutrient composition in neurodegenerative outcomes.^153^ Halagappa et al. reported that both IF and CR improved cognitive performance and reduced behavioral abnormalities in a triple‐transgenic AD mouse model.^154^ These dietary interventions show therapeutic potential by enhancing neuroprotection, decreasing Aβ deposition, and supporting neuronal health. Notably, IF increased brain‐derived neurotrophic factor levels, a critical neuroprotective protein that promotes neuronal health and may slow aging.^154^ Prolonged IF has also been shown to induce heightened yet functional macroautophagy and chaperone‐mediated autophagy in neurons, providing region‐specific neuroprotection against increased Aβ‐induced toxicity.^155^ Overall, IF and CR studies in mice models of AD have been shown to increase lifespan, which could be due to improved autophagic responses at both cellular and physiological level in alleviating neuronal damage.^129^, ^130^, ^131^ However, evidence from clinical trials and disease intervention studies in humans are still lacking.^129^, ^130^, ^135^ Moreover high‐fat and high‐calorie diets have been associated with impaired cognitive function and disrupted autophagy pathways. Specifically, a high‐fat diet suppresses mitophagy, leading to cognitive decline,^156^ while excessive calorie intake exacerbates age‐related hippocampal injury and alters autophagic activity.^150^ Furthermore, high‐cholesterol conditions impair lysosomal function, contributing to neurodegeneration, although long‐term exercise may mitigate these effects by enhancing brain lysosomal activity.^157^

#### 1.9.3 Human clinical trials

To date, there is no direct clinical evidence or trials linking IF‐induced neuronal autophagy to therapeutic outcomes in neurodegenerative diseases. However, peripheral studies suggest systemic benefits of autophagy activation via IF and CR. In a notable study of skeletal muscle, long‐term CR was associated with increased expression of key autophagy‐related genes (e.g., ATG101, APG12, Beclin‐1, GABARAP/GATE‐16, LC3, ULK1) in healthy adults.^158^ Participants (*n* = 111) were divided into three groups: CR practitioners (6 ± 3 years), endurance runners (21 ± 11 years), and sedentary controls. CR led to elevated autophagic markers, increased molecular chaperones, higher cortisol levels, and reduced inflammatory markers, suggesting systemic protein quality control benefits, though not directly in the brain.^158^ Evidence for IF in cognitive health is emerging. Other few studies reported improved cognitive function in older adults with mild cognitive impairment (MCI) who practiced alternate‐day fasting over 36 months compared to non‐fasters.^159^ Metabolic modulation, such as increased ketone production and glucose regulation, may underlie these effects. Two large observational studies in elderly Italians (*n* > 880) further linked IF with reduced cognitive impairment and mental distress.^160^, ^161^ However, smaller trials show mixed results. For instance, two small studies reported no significant cognitive effects of IF: one involving eight healthy males during Ramadan over a 2‐week period^162^ and another assessing overweight adults with mild to moderate functional limitations over 4 weeks.^163^ Overall, while fasting‐related strategies may promote autophagy and cognitive benefits, especially in at‐risk populations, large‐scale, well‐controlled human trials are needed to validate neuronal autophagy as a therapeutic mechanism in AD. Together, these studies firmly established autophagic dysfunction as a core feature of AD pathology, implicating impaired clearance of Aβ and tau as central drivers of neurodegeneration. However, despite substantial progress, the precise sequence of autophagy failure in AD – whether it originates upstream as a triggering event or emerges downstream from accumulating pathology – remains unresolved. Future research must therefore focus on defining the temporal dynamics of autophagy impairment in human neurons, mapping how genetic risk factors such as PSEN1 mutations disrupt lysosomal function and developing targeted strategies to restore autophagic flux with temporal and cell‐type specificity. Such work will be essential for determining whether autophagy enhancement can be safely and effectively leveraged as a disease‐modifying therapy in AD.

### 1.10 Ketogenic diet

#### 1.10.1 Mechanism of ketogenic diet and autophagy

Contrary to fasting, the ketogenic diet (KD), particularly the medium‐chain triglyceride (MCT) diet, is based on energy obtained from fats instead of gluconeogenesis. This in turn results in a high production of three main ketone bodies in the organism namely: acetone, β‐hydroxybutyrate (βHB), and acetoacetate, which brings about a state of ketosis.^164^, ^165^ This state of ketosis then induces autophagy in the brain through the inhibition of the mTORC1 complex and activation of hypoxia‐induced factor 1α (HIF‐1α) and sirtuin 1 (Sirt1).^166^, ^167^ Here, HIF‐1α activation induces BNIP3, BNIP3L, and BH3‐only proteins, which bind to Bcl‐2/Beclin‐1 complex, resulting in the disruption of the complex's inhibitory association^166^, ^167^ (Figure 2). Once this occurs, Beclin‐1 is then able to carry out its functional role in the initial step of autophagy, which is autophagosome formation. The activation of Sirt1 allows interaction and direct deacetylation with ATG proteins, which are then utilized in autophagy.^166^, ^167^ The inhibition of the mTOR pathway through the KD has been shown to be mediated by the reduction in pS6 and pAKT expression.^167^ Overall, KD aims to emulate the fasting condition in organisms without resulting in the negative effects of starvation.^166^

#### 1.10.2 Animal studies

The KD has shown promising beneficial effects in various cell and animal models of AD, particularly through the reduction of Aβ pathology. For example, in mouse hippocampal HT‐22 cells, KD delayed AD onset by reducing Aβ plaques via ketone‐dependent, mTOR‐mediated regulation of the HMGS2 enzyme involved in ketone synthesis.^168^ In transgenic AD mouse models, KD or ketone supplementation consistently reduced Aβ40 and Aβ42 levels. APP/V717I mice exhibited a 25% reduction of Aβ after 43 days of KD,^169^ whereas 5xFAD mice showed decreased Aβ deposition in hippocampus and cortex after 4 months of KD.^170^ In C57BL/6 mice, median survival and lifespan had significantly increased after 1 month of KD intervention,^171^ while daily ketone body injections in APP mutant mice reduced both soluble and insoluble Aβ42 and plaque burden over 2 months.^172^ In another study, 3xTg‐AD mice receiving ketone esters showed improved memory, reduced anxiety, and lowered Aβ and tau levels after 8 months.^173^ However, other studies report no effect of a KD on Aβ pathology despite improvements in motor or cognitive functions. In APP/PSEN1 transgenic mice, 16 weeks of a KD improved motor function but did not reduce Aβ deposition.^174^ Beckett et al. similarly found that a KD enhanced motor performance in APP/PSEN1 knock‐in mice but had no impact on brain or muscle Aβ levels or oxidative stress markers.^175^ Moreover, in non‐transgenic rats, a KD did not reduce Aβ deposition, unlike IF.^176^

Mechanistically, KD has been shown to activate autophagy‐related pathways in the brain (Table 1). For instance, 9‐week‐old male mice fed either of two KD formulations or standard chow for 4 weeks, increased LC3‐II levels and enhanced LC3 puncta formation were observed in the hippocampus and frontal cortex, indicating upregulated autophagosome synthesis.^177^ Similarly, C57BL/6N male mice fed a KD for 4 weeks exhibited increased Beclin‐1 and LC3‐II/LC3‐I ratio in the hippocampus, indicating autophagy activation.^178^ Additionally, in Sprague‐Dawley rats (epilepsy model), 2 weeks of KD feeding suppressed mTOR signaling, evidenced by decreased phosphorylation of mTOR, pS6, and pAkt in the hippocampus, which likely contributes to enhanced autophagic flux.^167^

Overall, the literature remains inconclusive regarding KD's ability to consistently reduce Aβ deposition and confer neuroprotection in AD models. Limitations include variability in KD regimes, animal models, and incomplete understanding of underlying biological pathways such as autophagy and mTOR signaling. Further studies are needed to clarify whether a KD can delay AD onset and progression by targeting Aβ pathology.

#### 1.10.3 Human clinical studies

Human clinical studies provide preliminary evidence supporting the potential of ketogenic interventions to improve cognitive function in patients with AD or MCI. Early trials using MCTs demonstrated acute improvements in memory correlating with elevated ketone body levels.^179^, ^180^ Longer‐term KDs supplemented with MCTs showed cognitive benefits that reversed upon cessation of the diet.^181^, ^182^ More recent randomized trials further suggest enhanced daily function and verbal cognition with carbohydrate restriction or ketogenic regimens in AD and MCI populations.^183^, ^184^

While these findings support the association between ketogenic metabolism and cognitive improvement, the underlying molecular mechanisms, particularly involving autophagy, remain to be fully elucidated. Notably, human trials have demonstrated more consistent cognitive benefits compared to animal studies, highlighting key anatomical, physiological, and behavioral differences between species. Despite shared gene homology, discrepancies in gene families, regulatory mechanisms, and physiological responses limit the direct translatability of animal findings to humans. A comprehensive, multilevel investigation, from molecular to systemic, is therefore essential to fully understand the therapeutic potential of KDs in AD.

### 1.11 Diets for AD prevention

Although extensively reviewed elsewhere, dietary patterns such as the Mediterranean, DASH, and Mediterranean‐DASH Intervention for Neurodegenerative Delay (MIND) diet are consistently associated with reduced AD risk, potentially through modulation of autophagy‐related pathways.^128^, ^185^, ^186^, ^187^ Beyond CR and ketogenic strategies, several nutritional components modulate autophagy and cognitive health. Omega‐3 fatty acids, particularly DHA, enhance neuronal resilience by activating AMPK and suppressing mTOR signaling and promoting autophagic flux.^188^, ^189^ Vitamins and micronutrients, including vitamin D, vitamin E, selenium, zinc, and B‐complex vitamins, support redox balance, limit tau hyperphosphorylation, and improve cognitive health.^190^ Polyphenols such as resveratrol (found in red grapes), EGCG (from green tea), curcumin (from turmeric), and oleuropein aglycone (from extra virgin olive oil) regulate autophagy through SIRT1, AMPK, and mTOR pathways, as well as histone acetylation and upregulation of autophagy‐related genes, enhancing clearance of misfolded proteins, supporting neuroprotection, and reducing oxidative and inflammatory injury.^191^, ^192^, ^193^, ^194^ These compounds likely contribute to the observed benefits of Mediterranean‐style dietary patterns in reducing AD risk. These mechanistic links suggest that autophagy may be a key mediator of the cognitive benefits observed with these diets, though human data confirming this relationship remain limited.

#### 1.11.1 Gut–brain axis, microbiota, and autophagy

The gut–brain axis plays a crucial role in regulating autophagy and AD progression through microbial metabolites, immune signaling, and neural pathways. Dysbiosis, or imbalance in the gut microbiota, promotes systemic inflammation and compromises blood–brain barrier integrity, facilitating Aβ and tau pathology.^195^, ^196^ Microbial metabolites such as short‐chain fatty acids (SCFAs), particularly butyrate, activate autophagy via the AMPK/SIRT1 pathway, enhance mitochondrial function, and support neuronal survival.^197^ Conversely, bacterial lipopolysaccharides (LPSs) activate microglia, suppress autophagy, and exacerbate oxidative stress, accelerating neurodegeneration.^198^

Diets rich in fiber, polyphenols, and prebiotics, including Mediterranean and MIND‐style dietary patterns, restore microbial diversity and strengthen autophagy‐related neuroprotection. These diets increase beneficial bacteria such as *Bifidobacterium* and *Lactobacillus*, which are associated with improved cognitive outcomes and reduced Aβ burden.^199^ Experimental evidence also indicates that probiotic supplementation can upregulate autophagy markers such as Beclin‐1 and LC3‐II, highlighting a bidirectional relationship between the gut microbiome and neuronal autophagy.^200^, ^201^ Collectively, these findings suggest that targeting the gut–brain–autophagy axis through dietary and microbial interventions may represent a promising therapeutic avenue for AD prevention and management.

Collectively, evidence from dietary intervention studies and gut–brain axis research suggests that nutrition exerts a powerful influence on autophagy, neuroinflammation, and cognitive resilience in AD. Mediterranean, DASH, and MIND dietary patterns – along with key nutrients such as omega‐3 fatty acids, polyphenols, and vitamins – appear to support neuronal health partly by activating autophagy‐related pathways and enhancing the clearance of misfolded proteins. Likewise, modulation of the gut microbiota through fiber‐rich foods, prebiotics, and probiotics may promote neuroprotection by restoring microbial balance, increasing SCFA production, and upregulating autophagy markers in both peripheral and central tissues. Despite these promising mechanistic links, direct clinical evidence confirming autophagy as the mediator of dietary benefits in humans remains limited. Future work should focus on well‐controlled longitudinal trials integrating molecular biomarkers of autophagy, microbiome profiling, and cognitive outcomes to clarify causality and identify targeted dietary strategies for AD prevention.

### 1.12 Physical activity and AD risk

Physical activity is well known to promote better health. Being physically active can improve heart and lung functions, promote brain health, help manage weight, reduce the risk of disease, strengthen bones and muscles, and improve the ability to do everyday activities. Exercise is a structured form of physical activity, and all exercises can be considered physical activity. Extensive reviews have discussed the benefits of physical activity in AD and the multiple biochemical pathways that mediate its protective effects.^202^, ^203^, ^204^

Several meta‐analyses showed that physical activity promoted cognitive function and ameliorated brain volume loss in individuals undergoing various exercise interventions, primarily aerobic exercises.^205^, ^206^, ^207^, ^208^, ^209^, ^210^ Of these studies, three meta‐analyses involved individuals who either had AD or MCI or were at risk of AD with a sample size ranging from 673 to 1145. Aerobic exercise significantly enhanced cognition, often measured by Mini‐Mental State Examination score, and increased cardiorespiratory fitness and hippocampal volume, correlating with better memory performance.^205^, ^206^, ^208^, ^211^ Resistance training also improved cognition, altered brain structure, and reduced white matter hyperintensities in older adults with MCI and early‐stage dementia.^212^, ^213^, ^214^ Both exercise types are beneficial, though no additive cognitive advantage has been observed from combining them.^205^ AD is a highly comorbid disease and generally co‐exists with multiple chronic conditions such as heart disease, stroke, type 2 diabetes, and high blood pressure. Because physical activity has a profound impact on overall health and helps to prevent and manage several chronic conditions, it is still yet to be determined whether cognitive benefits of different exercise regimes are dependent on the type of comorbidities observed in AD. Moreover, the etiologic role of physical activity in AD pathogenesis and fundamental mechanisms that reduce the risk of neurodegeneration remains to be fully understood.

Animal models of exercise are commonly used in research studies to investigate the beneficial effects of acute and chronic exercise conditioning on the brain and neurodegenerative diseases. In animal studies, particularly transgenic AD mouse models, it is now well recognized that exercise has substantial pro‐cognitive and pro‐neurogenic benefits.^215^ Using two main modes of exercise, treadmill running and voluntary wheel running protocols, studies have shown to significantly reduce AD pathology in transgenic mice brains by reduction in tau phosphorylation, soluble Aβ protein, and Aβ plaque levels.^216^, ^217^, ^218^ While the vast majority of the studies show the profound benefits of physical activity in AD, some studies have shown no significant effect of exercise on amyloid pathology.^219^ This, therefore, highlights a need for further research in understanding the mechanisms involved in which exercise lowers AD and amyloid pathology. In the next section, we summarize the experimental evidence of physical activity and its impact on autophagy in AD models and further discuss the possible mechanisms of action based on recent advances.

#### 1.12.1 Effects of physical activity on autophagy in AD

**FIGURE 3:**
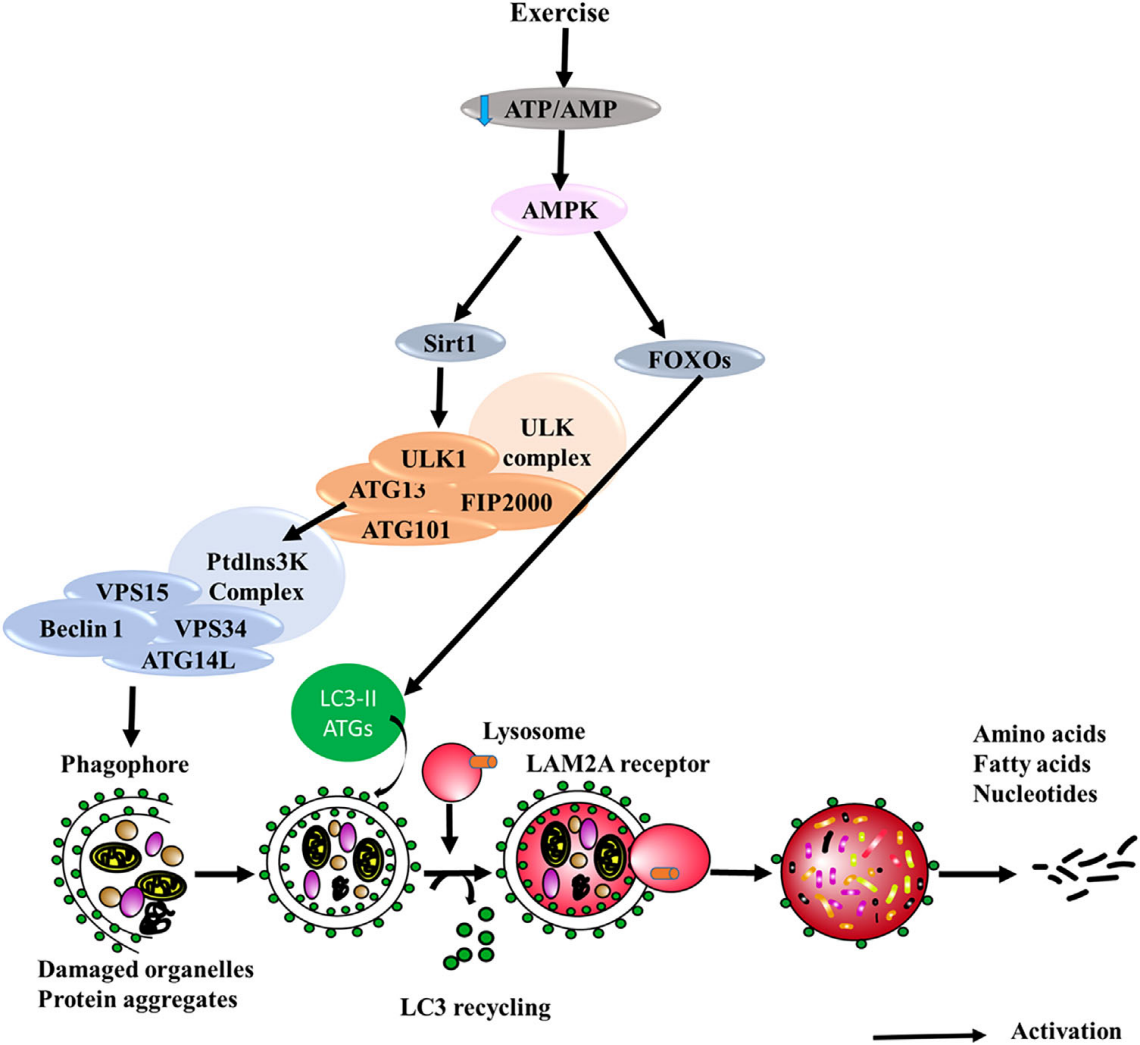
Impact of exercise on modulation of autophagic pathway. Exercise stimulates autophagy by activating pathways that include ULK1 and SIRT1, which deacetylates and activates FOXO3, thereby boosting autophagy via upregulation of autophagy‐related genes. Collectively, these lifestyle modifications, like exercise, regulate autophagy, which is essential for preserving cellular integrity and averting age‐related disorders, including neurodegenerative diseases. AMP, adenosine monophosphate; AMPK, adenosine monophosphate‐activated protein kinase; ATG, autophagy regulated genes; ATP, adenosine triphosphate; ULK1, Unc‐51 like autophagy activating kinase 1; mTOR, mechanistic target of rapamycin.

Exercise generally enhances autophagy and reduces neuropathology in animal models of AD, although the exact mechanism is still unknown. PI3K/Akt, Wnt/β‐catenin, AMPK‐ULK1, MAPK, NF‐κB, PINK1‐PARKIN, JAK/STAT, and TREM2 are among the autophagy‐related signaling pathways that are activated by exercise. These pathways collectively affect autophagic flux, mitophagy, lysosomal function, mitochondrial homeostasis, and inflammation.^220^ Exercise is believed to promote autophagy, which is similar to dietary interventions and helps to improve neuronal homeostasis and Aβ clearance.^157^, ^221^, ^222^, ^223^, ^224^, ^225^ In addition to improving mitochondrial quality control and lowering apoptosis, moderate treadmill exercise frequently raises LC3‐II and decreases p62/SQSTM1, which is consistent with increased autophagic flux.^201^, ^202^, ^203^, ^204^, ^226^, ^227^, ^228^, ^229^, ^230^, ^231^, ^232^, ^233^, ^234^, ^235^ According to more recent research, exercise can help lower Aβ pathology and cognitive deficits by activating specific autophagy pathways, such as ER‐phagy through FAM134B and lysosomal enhancement via PGRN.^227^ Exercise also seems to stimulate mitophagy, which is facilitated by the PINK1/Parkin and SIRT1‐FOXO1/FOXO3 axis and results in increased mitochondrial turnover and decreased Aβ accumulation.^228^, ^229^ Further supporting the wider control of autophagy and lysosomal biogenesis is the activation of AMPK, SIRT1, TFEB, Beclin‐1, and LC3 brought on by exercise^230^, ^231^, ^232^, ^233^, ^234^, ^235^ (Figure 3). The idea that exercise alters AD pathology by activating conserved autophagy and proteostasis pathways is supported by these studies taken together.^236^, ^237^, ^238^, ^239^

Findings are still mixed in spite of these encouraging patterns. Following exercise, a number of studies find no discernible changes in LC3 or p62 in different parts of the brain,^223^, ^240^, ^241^, ^242^ while others even report drops in both markers.^157^ These disparities most likely result from variations in the transgenic model, baseline autophagy impairment, duration (short‐term vs chronic), intensity (moderate vs exhaustive), exercise type (aerobic, resistance, endurance), and the timing of tissue collection following the last exercise session (Table 2). Crucially, autophagy is a dynamic and circadian‐modulated process, and misleading conclusions can be drawn if static markers (LC3, p62) are used without evaluating lysosomal function or flux. Mechanistic interpretation is limited by the lack of autophagy flux assays, lysosomal activity measurements, and regional comparisons throughout the CNS in exercise studies.

**TABLE 2:** Autophagy‐related expressions in brain regions following exercise interventions.

Exercise type	Study	Mouse model	Age at beginning of intervention	Exercise duration	Tissue collection latency from last intervention	AD‐ and autophagy‐related expressions of interest findings
Treadmill	Zhao et al.^273^	APP/PSEN1	3 months	12 weeks	Hippocampus	↑ LC3II; ↓ p62, LAMP1, Aβ40, Aβ42
Running wheel	Wang et al.^157^	APP/PSEN1	5 months	5 months	Hippocampus and cortex	↑ m‐CatD, m‐CatL, Atg5‐Atg12, TFEB, pULK1Ser555, AMPK; ↓ LC3II, p62, LAMP1, TauSer396, Bctf,
Treadmill	Kang & Cho^222^	Nse/htau23	18 months	12 weeks	Cerebral cortex	↑ LC3B, p‐P13K/t‐PI3K, p‐AKT/t/AKT, Beclin‐1; ↓ p62, p‐mTOR/t‐mTOR, Ser199/202, Ser404, Thr231, PHF1
Treadmill	Broderick et al.^223^	3xTg‐AD	8 weeks	5 weeks	Hippocampus and cerebral cortex	↓ LC3I, LAMP2, CatB
Treadmill	Zhao et al.^229^	APP/PSEN1	3 months	12 weeks (five times of 45 min exercise per week)	Hippocampus	↓ PINK1; ↑ Parkin; ↓ P62, A β peptide; ↑ LC3II, SYN, GAP43, PGC‐1 α, TFAM
Treadmill	Zhao et al.^228^	APP/PSEN1	3 months	12 weeks (five times of 45 min exercise per week)	Hippocampus	↓ PINK1, Ace‐FOXO1a (Lys294), Ace‐FOXO3a (Lys271); ↑ Parkin, SIRT1; ↓ P62, Aβ40, Aβ42; ↑ LC3II/I,
Treadmill	Ohia‐Nwoko et al.^240^	P301S	7 to 8 months	12 weeks	Hippocampus and cortex	↓ TAU5, AT8, AT100, AT180
Running wheel	Gratuze et al. (2017)^241^	hTau	6 months (2 months at start of diet diversity)	2 months	Hippocampus	∼LC3II, LC3I, LAMP1, Atg5, Atg9a; p‐AMPKa, AMPKa
Treadmill	Koo & Cho^236^	C57BL/6 and C57BL/6 MPTP/P treated (MPTP and probenecid) [PD mouse model]	7 weeks	8 weeks	Complete brain (1 week after last exercise)	↓ p62
Treadmill	Jang et al.^237^	C57BL/6, and C57BL/6 MPTP/P treated	7 weeks	8 weeks	Substantia nigra (1 week after last exercise)	↑ LC3II, Beclin‐1, Bcl2; ↓ p62
Motorised wheel	Zhang et al.^238^	Sprague‐Dawley MCAO model		1, 2, 3 weeks	Complete brain (immediately after exercise)	↓ LC3
Treadmill	Li et al.^239^	C57BL Type 2 diabetes mellitus model	4 weeks	8 weeks	Hippocampus (36 h after last exercise)	↑ LC3B, LC3II/LC3I Beclin‐1; ↓ p62, p‐mTOR/mTOR, AKT
Treadmill	Minakaki et al.^242^	aSyn knockout mice (KO Syn), C57BL6N (WT)	7 to 8 months	4 weeks	Cerebral cortex (16 h after last exercise)	∼ LC3II, p62, LAMP2A, pS6/S6
Treadmill	Huang et al.^231^	EX527 injected		Long‐term: 8 weeks; Short term: 1 h	Cerebral cortex, hippocampus, and striatum (after 18 rpm (14:00 to 17:00), 5 days/week for 8 weeks)	**Long‐term exercise**: ↑ LC3II, LAMP1, TFEB, m‐CatD, m‐CatL; ↑ LAMP1, TFEB, m‐CatD, mCatL; ↑ (Striatum) LC3II, LAMP1, TFEB, m‐CatD, m‐CatL; ∼ LC3II; **Short‐term exercise**: Expressions fluctuate over time
Treadmill and running wheel	Rocchi and He^230^	C57BL/6	8–12 weeks	4 days, and 2 weeks	Treadmill tissue collected after exercise; Wheel tissue collected a day after exercise	↑ LC3
Treadmill	Jang^234^	C57BL/6J	7 weeks (male)	6 weeks	Hippocampus (2 h after last exercise)	↑ LC3II, p62, LAMP2, TFEB, CatL, Atg7, p‐mTOR/mTOR, p‐AKT/AKT, p‐ULK1ser555/ULK1, Beclin‐1, p‐BCL‐XL/BCL‐XL, p‐AMPK/AMPK, p‐P70S6K/P70S6K
Treadmill and running wheel	Marques‐Aleixo et al.^232^	Sprague‐Dawley	21 days	12 weeks	Cerebral cortex and cerebellum (48 h after last exercise)	**Treadmill**: ↑ Beclin‐1, Beclin‐1/Bcl2, LC3II; **Wheel**:↑ Beclin‐1, LC3II, Beclin‐1/Bcl2; **Treadmill & Wheel**: ∼ (Cortex and Cerebellum) p62
Treadmill	Kwon et al.^233^	C57BL/6	7 weeks (when received), 10 weeks when conducted	6 weeks	Cerebral cortex (1 h after last exercise)	↑ LC3II, p62, TFEB, LAMP2, CatL, Atg7, p‐mTOR/mTOR, p‐AKT/AKT, p‐ULK1Ser555/ULK1Ser555, Bcl2, p‐AMPK/AMPK, p‐P70S6K/P70S6K
Treadmill	Liu et al.^235^	Sprague‐Dawley	3, 16, and 23 months	10 weeks	Hippocampus (1 day after last exercise)	↑ LC3II, Beclin‐1, AMPKa1
Aerobic exercise	Liu et al.^225^	Sprague‐Dawley	13 months	10 weeks	Hippocampus	↑ LC3, Beclin‐1, pAMPKa1/AMPKa1

*Note*: ↑, increased expression in autophagic markers, ↓, decreased expression in autophagic markers, ∼, no significant difference in expression of autophagic markers.

Abbreviations: Aβ, amyloid beta; AD, Alzheimer's disease; AMPK, adenosine monophosphate‐activated protein kinase; TFEB, transcription factor EB.

Overall, research indicates that while prolonged or high‐intensity exercise may cause oxidative or metabolic stress that interferes with autophagosome‐lysosome fusion, moderate, sustained physical activity most consistently increases autophagic flux through AMPK‐ULK1 and SIRT1‐PINK1/Parkin signaling.^207^, ^226^, ^239^ Significant knowledge gaps still exist, though, including (1) establishing exercise dose–response thresholds for optimal autophagy; (2) comprehending the ways in which age, sex, and model‐specific pathology affect autophagic responsiveness; (3) identifying the selective autophagy pathways (ER‐phagy, mitophagy) that are most pertinent for AD modification; and (4) elucidating whether autophagy induction serves as a direct mediator of cognitive benefits or through interrelated vascular, metabolic, or inflammatory effects. Standardized exercise paradigms, exacting autophagy flux measurements, and the incorporation of human cell‐based models to increase translational relevance will all be necessary to close these gaps. A more extensive analysis is warranted to determine the specific autophagy changes in the CNS of animal models that take place at different ages and the extent to which type of exercise and duration alter the pathway to reduce AD neurodegeneration.

### 1.13 Sleep quality, brain health, and AD

Sleep is a highly conserved biological process essential for metabolic homeostasis, cognitive function, and overall health. Sleep undergoes changes with aging and lifestyle factors,^243^, ^244^ and its poor sleep quality is linked to neurodegenerative diseases and other chronic illnesses, including hypertension, diabetes, and stroke.^245^, ^246^, ^247^ Research indicates that sleep quality, including continuity, efficiency, and depth, is an important predictor of neurological health and AD risk.^248^ Poor sleep quality, even without total sleep loss, has been linked to impaired cognition and decreased glymphatic clearance of metabolic waste.^249^ Recent research has also shown that poor sleep quality increases the risk of AD,^248^ implying that fragmented or non‐restorative sleep may be just as harmful as short sleep duration. A common feature of many sleep disorders is decreased sleep quality, which reflects not only total sleep time but also the consistency of sleep continuity, sleep depth, and the integrity of rapid eye movement (REM) and non‐REM cycles. Insomnia and sleep fragmentation primarily disrupt sleep continuity, while circadian rhythm disorders impair sleep timing and architecture, all of which contribute to poor restorative sleep quality. These disturbances degrade sleep quality by impairing memory consolidation, altering neurotransmitter balance, and decreasing the efficiency of glymphatic and autophagy‐mediated clearance pathways.^249^, ^250^, ^251^ Thus, sleep quality serves as a unifying concept that connects various sleep disorders to shared mechanisms of brain disorders, providing a coherent foundation for understanding how sleep disruption, whether through deprivation, fragmentation, or REM loss, compromises proteostasis and increases the risk of AD.

According to the International Classification of Sleep Disorders, sleep disorders include insomnia, hypersomnolence, circadian rhythm disorders, parasomnias, and sleep‐related movement disorders.^252^ Sleep deprivation and sleep fragmentation are important components of sleep disorders and are associated with anxiety, poor cognition, and impaired brain performance.^28^, ^143^, ^253^ Sleep plays a crucial role in memory processing and neurological health, primarily by regulating a wide array of neuroanatomical and neurochemical systems, including acetylcholine, dopamine, noradrenaline, serotonin, histamine, and hypocretin.^254^, ^255^

Sleep is a key component for better brain maintenance and health, and sleep disturbances are associated with an increased risk of developing AD. A meta‐analysis of 27 observational studies found a 1.68‐fold increased risk of AD and cognitive impairment in individuals with sleep difficulties.^256^ Multiple studies have demonstrated the effect of sleep deprivations on AD pathologies like Aβ and tau accumulations in both animal models and humans.^257^, ^258^, ^259^ Both sleep deprivation and sleep fragmentation have been shown to increase Aβ and tau accumulations by dysregulating autophagy mechanisms in AD mouse models.^260^, ^261^, ^262^, ^263^, ^264^ Approximately 45% of AD patients report sleep disturbances, suggesting a bidirectional relationship between sleep and AD.^265^, ^266^ Sleep deprivation often occurs before cognitive decline, making it a potential early biomarker and therapeutic target.^267^, ^268^, ^269^, ^270^, ^271^

A cross‐sectional study on 4425 cognitively unimpaired participants showed an increased risk of Aβ deposition with reduced night‐time sleep.^272^ Even one night of sleep deprivation raises chronic sleep deprivation Aβ levels by over 30%,^222^, ^273^ and partial chronic sleep deprivation elevates cerebrospinal fluid orexin, a regulator of sleep–wake cycles.^274^, ^275^ Other systematic reviews further confirm sleep disturbances significantly raise dementia risk.^276^, ^277^

Animal models demonstrate that sleep deprivation exacerbates memory loss, synaptic damage, and tau pathology while also increasing insoluble tau and glial activation markers.^258^ More recent evidence has shown that sleep has a direct role in regulating the clearance of metabolic waste, including Aβ, from mice brains.^278^ The close link between AD pathogenesis and sleep deprivation is further supported by findings showing that Aβ and tau commonly accumulate in the suprachiasmatic nucleus and lateral hypothalamic areas of brain regions critically involved in the regulation of sleep–wake cycles.^279^ While mounting evidence implicates disturbed sleep or lack of sleep as a risk factor for AD, the etiologic role of sleep in AD pathogenesis and neuroprotective pathways associated with sleep intervention remains to be fully understood.

### 1.14 Sleep dysfunction and autophagy

**FIGURE 4:**
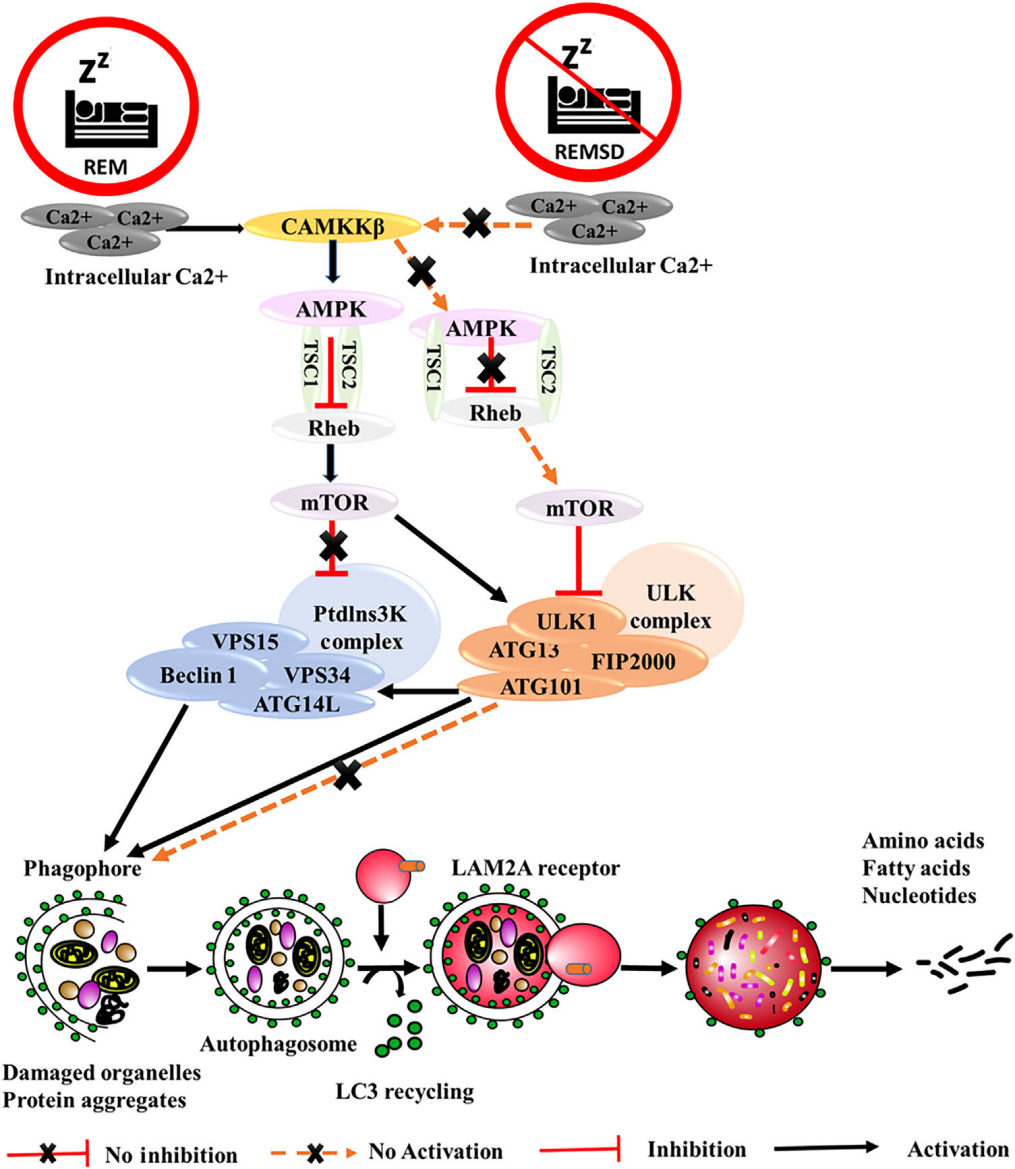
Impact of lifestyle intervention such as sleep on modulation of autophagic pathway. During REM sleep, variations in intracellular Ca^2^^+^ concentrations activate Ca^2+^/CaMKKβ, which in turn promotes AMPK to inhibit mTOR, thereby promoting autophagy. During REM sleep deprivation, reduction in Ca^2^^+^ concentrations reduce autophagy via mTOR. These lifestyle modifications maintain and regulate autophagy, which is essential for preserving cellular integrity and averting age‐related disorders, including neurodegenerative diseases. AMPK, adenosine monophosphate‐activated protein kinase; ATG, autophagy regulated genes; CaMKKβ, calmodulin‐dependent protein kinase β; mTOR, mechanistic target of rapamycin; REM, rapid eye movement; ULK1, Unc‐51 like autophagy activating kinase 1.

Building upon the established association between sleep quality and AD risk, emerging evidence highlights that sleep also directly regulates autophagic activity and protein homeostasis in the brain. Like autophagy, a cellular clearance pathway, sleep serves as a physiological process that clears unwanted and accumulated proteins from the brain and other parts of the body, thereby maintaining protein homeostasis.^3^, ^280^ Sleep cycles or circadian rhythms play a prominent role in regulating clearance and the protein homeostasis mechanism.^280^, ^281^ Mammalian sleep comes in two electrophysiological and metabolic states. REM sleep is observed in mammals and birds and associated with high brain‐metabolic demands, nearly indistinguishable from wakefulness.^264^, ^282^ REM sleep is key for new memory formation and a healthy brain. REM sleep is known to be altered in most of the neurodegenerative diseases.^264^, ^283^ Furthermore, REM sleep deprivation is known to affect brain excitability, neurogenesis, synaptic pruning, and memory consolidation^264^ (Figure 4).

Animal studies show that REM sleep is necessary for maintaining protein homeostasis and promoting autophagy via AMPK phosphorylation, which activates the TSC1/TSC2 complex and inhibits mTOR activity to initiate autophagosome formation.^262^, ^264^ Disturbances in REM sleep disrupt Ca^2^^+^‐dependent CaMKKβ‐AMPK signaling, dysregulating autophagy and increasing vulnerability to neurodegeneration.^247^, ^264^, ^284^ Because sleep and circadian rhythms are closely linked, autophagy shows significant diurnal variation: Circadian genes peak during the sleep phase and upregulate autophagy‐related transcription, enhancing metabolic waste clearance and maintaining proteostasis.^143^, ^147^, ^232^ Disrupted sleep thus influences both the activation of autophagy pathways and the timing of their circadian regulation.

In animal models, poor sleep quality, including sleep fragmentation and sleep deprivation, consistently impairs autophagic flux and lysosomal function.^285^ Fragmented sleep disrupts the endosome—autophagosome–lysosome axis, alters LC3 cycling (Table 3), and suppresses normal circadian oscillations of autophagy proteins in the hippocampus, indicating early neurodegenerative dysfunction.^260^, ^263^, ^286^ Mitochondrial quality control is also impacted, with mitophagy pathways such as PINK1/Parkin shifting based on metabolic demand between sleep and wake states,^287^ linking sleep disruption to impaired proteostasis. Furthermore, two‐photon imaging has revealed that autophagosome formation follows a circadian pattern independent of feeding behavior, confirming sleep's regulatory control over neuronal autophagy.^138^ Sleep deprivation has far‐reaching consequences for the body (Table 3). Sleep deprivation activates AKT/mTOR signaling in the liver and suppresses autophagy,^288^ whereas obstructive sleep apnea causes oxidative stress, endothelial dysfunction, hypoxia, and ER stress, all of which can lead to maladaptive autophagy responses.^289^, ^290^, ^291^, ^292^ Sleep‐deprivation‐induced thyroid injury has also been linked to imbalanced autophagy and apoptosis,^288^ though whether these peripheral autophagy abnormalities contribute to AD pathology is unknown.

**TABLE 3:** Autophagy‐related expressions in brain regions following sleep deprivation conditions.

Sleep disorders	Study	Mouse model	Techniques	Age/group	Brain region	AD‐related interest findings and gene activations
5 days of sleep fragmentation	Cheng et al.^263^	C57BL/6 mice	Western blot, RNA extraction, reverse transcription, qPCR	10 to 12 weeks old	Striatum, hippocampus, and frontal cortex	↑ (Striatum) Beclin‐1, LC3II, and p62; ↓ LAMP1 and TFEB; ↓ (hippocampus) Beclin‐1, TFEB, and p62; ↑ LC3II
Sleep deprivation	He et al.^260^	C57/B6 mice	Western Blot and immunofluorescence	8 to 12 weeks	Hippocampus	↓ LC31 and LC3II; ↓ LC3II/LC3I ratio; ↑ Beclin‐1
Chronic sleep fragmentation	Xie et al.^286^	C57BL/6J	Electron microscopy, immunofluorescence, and Western blot	2 months	Cortex and hippocampus	↑ Intracellular Aβ, UVRAG, and Beclin‐1 (cortex and hippocampus); ↑ microglial activation, ↑ Rab5, Rab7, and LC3B
Chronic sleep deprivation	Qiu et al.^285^	APPswe/PS1E9	Immunoblotting, immunostaining, electron microscopy, TUNEL assay	4 to 4.5 months old	Cortex and hippocampus	↑ ptau; ↑ mitochondrial damage, caspase cascade activation, neuronal apoptosis (hippocampus); ↑ Aβ1‐42, and senile plaques (cortex and hippocampus)
4 h sleep restraint per day for 8 weeks	Meco et al.^258^	3xTg‐AD	Immunoblotting, Immunohistochemistry	8 months	Brain cortex homogenates	↓ Learning and memory; ↓ tau phosphorylation; ↑ insoluble tau fraction; ↓ postsynaptic density protein 95; ↑ glial fibrillary acidic protein
Sleep deprivation for 72 h	Gao et al.^300^	Wistar rats	Y‐maze, Novel Object Recognition, Object location, and Morris water maze tests, Western blot analysis		Hippocampus	Sleep deprivation ↑ Beclin‐1 and ↓ p62 Sodium hydrosulfide treatment ↓ Beclin‐1 and ↑ p62
96 h of sleep deprivation	Dai et al.^297^	Male Sprague‐Dawley rats	Transmission electron microscopy, Western blot, PCR, immunohistochemistry	9 to 12 weeks	Hippocampus	↓ Beclin‐1, PINK1, parkin, p62, and LC3

*Note*: ↑ indicates increased expression in autophagic markers, ↓ indicates decreased expression in autophagic markers.

Abbreviations: Aβ, amyloid beta; AD, Alzheimer's disease; qPCR, quantitative polymerase chain reaction; TFEB, transcription factor EB.

Pharmacological sleep modulators provide additional mechanistic insight. Melatonin, a key circadian regulator, promotes autophagy by activating AMPK and mTOR‐dependent pathways, providing neuroprotection in AD models.^293^, ^294^ Pinocembrin promotes BNIP3‐mediated mitophagy during intermittent hypoxia,^295^, ^296^ whereas propofol appears to suppress excessive autophagy and mitophagy during sleep deprivation stress, improving cognitive outcomes.^297^ However, propofol's effects are likely to involve both autophagy modulation and restoration of GABAergic excitatory/inhibitory balance, emphasizing the difficulty of interpreting its mechanisms.^298^ Nonetheless, considering that propofol predominantly functions as a GABA receptor agonist and that the concentrations necessary for GABAergic activation are significantly lower than those affecting autophagy, its cognitive advantages may also pertain to the reestablishment of the excitatory/inhibitory (E/I) equilibrium. Recent evidence indicates that the GABAergic system, the principal inhibitory neurotransmitter network and a crucial regulator of excitatory/inhibitory balance, is significantly disrupted in AD, exacerbating synaptic dysfunction and disease advancement.^299^ Thus, the beneficial effects of propofol may result from a combination of autophagy regulation and correction of GABAergic dysfunction underlying E/I imbalance in AD. Additional modulators, including hydrogen sulfide, suppress pathological autophagy and improve cognitive function in sleep‐deprived animals.^300^ Notably, autophagy responses to sleep modulation appear to be state‐dependent: Sleep induction promotes autophagic flux in physiological conditions, whereas sleep deprivation has varying effects depending on circadian timing and cellular stress.^301^ Although these studies show neuroprotective actions of sleep modulators, their mechanisms of action and specific targets need to be validated, and, more importantly, how they modulate autophagy to protect against AD neurodegeneration still needs to be elucidated.

These findings suggest that sleep, specifically sleep quality and REM integrity, plays an important role in regulating autophagy, mitophagy, and protein clearance. Although, these studies show neuroprotective actions of sleep modulators, their mechanisms of action and specific targets need to be validated and more importantly how they modulate autophagy to protect against AD neurodegeneration still needs to be elucidated. However, significant knowledge gaps exist regarding the relative contributions of different sleep stages, the directionality of sleep–autophagy interactions in early AD, and the extent to which peripheral autophagy alterations influence CNS pathology. These uncertainties highlight the need for additional mechanistic studies, ideally combining circadian biology, autophagy flux measurements, and AD‐relevant models, to better understand how improving sleep quality can restore proteostasis and reduce neurodegeneration.

## 2 DISCUSSION

There is considerable evidence that a healthy lifestyle contributes to reduced dementia risk and improved metabolism through cardiovascular and cerebrovascular mechanisms. At a cellular level, lifestyle interventions are known to alter a wide range of pathways, including oxidative stress, inflammation, neurogenesis, anti‐aging/cell death, and clearance mechanisms such as autophagy. Autophagy is dysregulated in AD brains, and evidence shows that this pathway is closely connected and interlinked with diet, physical activity, and sleep‐related pathways and regulatory genes. Although clinical observations demonstrate that healthy lifestyles reduce dementia risk, our knowledge of the cellular mechanisms is largely derived from animal models, especially mice. Diet through fasting, improved sleep cycles, and exercise are all reported to induce autophagy in animal models. Increased expression of autophagy pathway‐related proteins, mainly LC3, has commonly been associated with reduced neuropathology in these studies. However, some studies show no effects on autophagy pathway proteins, and others report detrimental effects due to overactivated autophagy exacerbating AD pathology. Overall, autophagic response seems to vary significantly depending on type of health intervention, duration of intervention, animal model, and treatment age. Autophagy is a dynamic process altering across the day and night, between fasting and fed states, and during physical activity. Also, autophagy varies naturally between different types of cells, tissues, and organs depending on the physiological state of the organism. A balanced autophagic pathway is essential for cellular homeostasis, preventing the accumulation of toxic aggregates while avoiding excessive self‐digestion. Excessive autophagy causes cell death, while defective autophagy leads to toxic buildup, contributing to various neurodegenerative diseases Therefore, targeting autophagy through therapeutic interventions could offer promising strategies for age‐related and pathological conditions.^302^

Collectively, dietary composition, physical activity, and sleep quality interact to maintain autophagic balance and neuronal homeostasis. Each factor influences shared nutrient‐sensing and energy‐regulating pathways – such as AMPK, mTOR, and SIRT1 – as well as circadian and redox signaling mechanisms. Adequate nutrient intake and polyphenol‐rich diets enhance autophagic flux, regular exercise promotes mitophagy and lysosomal turnover, and consolidated sleep sustains circadian autophagy rhythms that coordinate proteostasis. These converging mechanisms underscore the notion that lifestyle factors act synergistically rather than independently, supporting the concept that holistic behavioral modification may provide optimal neuroprotection against AD.

**FIGURE 5:**
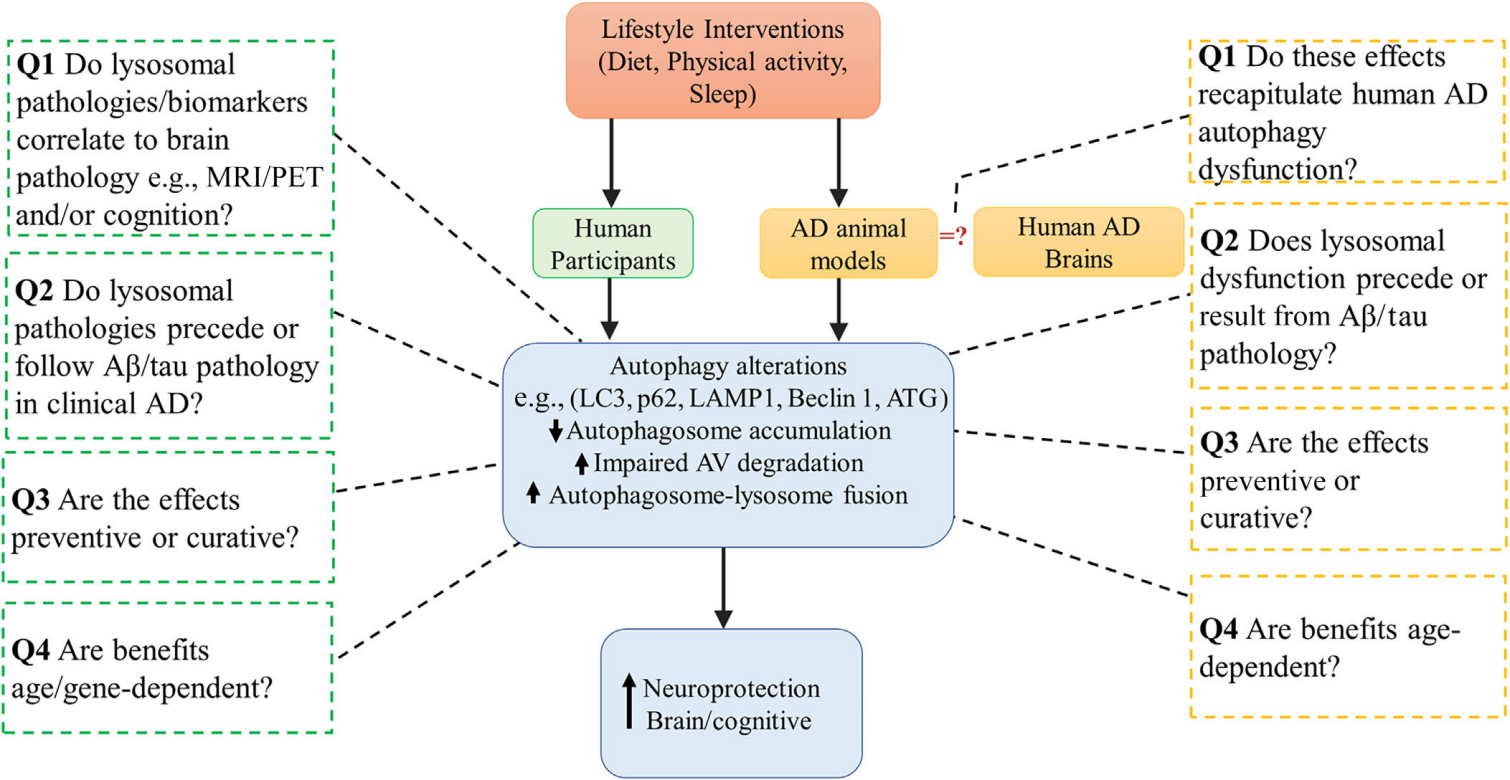
Key research questions regarding relationship between lifestyle interventions and autophagy dysfunction. This figure illustrates important research questions regarding relationship between lifestyle interventions (diet, exercise, and sleep) and autophagy dysfunction. AD, Alzheimer's disease; Aβ, amyloid beta; MRI, magnetic resonance imaging; PET, positron emission tomography.

Despite numerous reviews, there remains significant uncertainty around how best to evaluate autophagy, especially in model organisms like mice. Autophagy guidelines recommend the use of multiple complementary techniques and markers that reflect distinct stages of the pathway, rather than relying solely on endpoints like LC3 or p62.^303^ This methodological complexity is further compounded by the well‐recognized limitations in translating findings from mice to humans, due to both biological divergence and differences in how experimental interventions affect each species. Improving our understanding of lifestyle‐induced autophagy in AD will require more refined models that better reflect human biology. Emerging technologies such as human stem cell‐derived neurons and brain organoids will offer promising platforms to dissect how dietary and lifestyle interventions influence autophagy dynamics in human cells under aging and neurodegenerative conditions.^304^ These models may help bridge the gap between mechanistic insight and clinical relevance, particularly where current mouse models are lacking. Growing evidence in the autophagy field has also revealed that endpoint measurement of the gene and protein levels of markers such as LC3 and p62 may be misinterpreted and not reflective of changes in a dynamic system such as autophagy. Although LC3 increase could mean upregulation of autophagy, this may also indicate impaired lysosomal fusion, which may be related to reduced autophagic flux, or it could be an indicator of increased autophagosome formation.^305^, ^306^ AD is linked with the disturbances of the autophagy‐lysosomal pathway and endosomal trafficking.^64^ Electron microscopy examinations show an increased buildup of LC3‐containing autophagosomes in the cortex of the brain occurring across diverse phases of AD. In AD, autophagosomes accumulate owing to the overstimulation of autophagy, combined with a failure in autophagosome–lysosome fusion to enable recycling and degradation of the unwanted cell material including protein aggregates.^307^ Considering the preexisting lysosomal dysfunction and overactivation of autophagy in the AD brain, the observed changes in autophagy markers, whether increased or decreased, in AD mouse models subjected to various lifestyle interventions raises important questions (Figure 5). Although studies investigating autophagy dynamics in the human AD brain remain limited, the emerging data prompt several critical questions that warrant further exploration in future research (Figure 5).

With an aging population worldwide and most AD cases being of sporadic origin, lifestyle interventions present a safe, effective, and widely accessible approach for reducing dementia risk. The WHO has developed recommendations, including several cognitive, behavioral, social, and pharmacological interventions, aimed at improving health overall and reducing risk factors associated with cognitive decline and dementia.^308^ Researchers even suggest that genetic risk can be offset by lifestyle factors. In individuals with low genetic risk, modifiable‐risk profiles were related to a lower risk of dementia.^309^ It is, therefore, of interest to identify AD risk genes that are associated with lifestyle factors to guide better understanding of the AD pathological cascade, improve accuracy of prediction of disease onset through protein and gene biomarker analyses, and, most importantly, inform intervention trials for AD prevention. Despite their critical importance in translation into public health strategies, lifestyle interventions have several limitations and challenges in study design, implementation, and finding interpretation in the context of their impact on disease pathways and cellular mechanisms. This is mainly owing to the complex nature of multidomain lifestyle interventions, multitarget effects, socioeconomic factors, and diversity in cultures and behaviors. Furthermore, baseline status, finding appropriate control groups, blinding, randomization, and poor adherence can negatively impact the effectiveness of such trials. Prior studies provided some guidelines for formulating effective protocols to achieve the best effects of autophagy, but there are currently no reliable biomarkers to measure brain autophagy to determine thresholds and how to implement the different lifestyle changes to gain those benefits in a safe and sustainable manner. Future research should, therefore, focus on establishing biomarkers for measuring autophagy, determine safety thresholds for autophagy activation, and evaluate the independent and synergistic benefits of diet, regular physical activity, and sleep patterns. For the establishment of clinical guidelines, future trials will also need to employ larger sample sizes with longer follow‐ups and maximize participants’ adherence and accessibility.

## 3 CONCLUSION

Lifestyle factors such as diet, physical activity, and sleep all influence autophagy and contribute to a lower risk of AD, but the underlying mechanisms are only partially understood. While preclinical research consistently shows that fasting, exercise, and circadian‐regulated sleep can improve autophagic flux and proteostasis, translation to humans is hampered by model‐specific differences, reliance on static markers such as LC3 or p62, and a lack of reliable biomarkers for brain autophagy. The complexity of autophagy, including its circadian oscillation, cell‐type specificity, and susceptibility to both insufficient and excessive activation, suggests that lifestyle interventions may have highly context‐dependent effects in aging and AD. Future research should focus on developing sensitive, non‐invasive autophagy biomarkers, using human‐relevant systems like induced pluripotent stem cell‐derived neurons and brain organoids, and designing long‐term, culturally adaptable lifestyle intervention trials with higher adherence. Integrating genetic risk stratification with multidomain behavioral interventions could lead to personalized, evidence‐based strategies for safely modulating autophagy and promoting healthy brain aging.

## AUTHOR CONTRIBUTIONS

Prashant Bharadwaj conceptualized the study. Prashant Bharadwaj, Ajish Ariyath, Zoe Mputhia, Christopher Dougherty, and Bushra Kaleelur Rahuman drafted the manuscript. W. M. A. D. Binosha Fernando, Stephanie R. Rainey‐Smith, Belinda Brown, Samantha L. Gardener, and Ralph Martins reviewed the manuscript.

## CONFLICT OF INTEREST STATEMENT

The author(s) declare no competing financial or non‐financial interests. Author disclosures are available in the Supporting Information.

## Supporting information



Supporting information
